# Antiulcer Potential of *Psidium guajava* Seed Extract Supported by Metabolic Profiling and Molecular Docking

**DOI:** 10.3390/antiox11071230

**Published:** 2022-06-23

**Authors:** Nourhan Hisham Shady, Hend Samy Abdullah, Sherif A. Maher, Amgad Albohy, Mahmoud A. Elrehany, Fatma Alzahraa Mokhtar, Hesham Farouk Oraby, Ahmed M. Shawky, Usama Ramadan Abdelmohsen

**Affiliations:** 1 Department of Pharmacognosy, Faculty of Pharmacy, Deraya University, Universities Zone, New Minia City 61111, Egypt; norhan.shady@deraya.edu.eg; 2 Faculty of Pharmacy, Deraya University, Universities Zone, New Minia City 61111, Egypt; hend.samy_1180443@student.deraya.edu.eg; 3 Department of Biochemistry, Faculty of Pharmacy, Deraya University, Universities Zone, New Minia City 61111, Egypt; sherif.ali@deraya.edu.eg (S.A.M.); mahmoud.elrehany@deraya.edu.eg (M.A.E.); 4 Department of Pharmaceutical Chemistry, Faculty of Pharmacy, The British University in Egypt, Cairo 11837, Egypt; amgad.albohy@bue.edu.eg; 5 The Center for Drug Research and Development (CDRD), Faculty of Pharmacy, The British University in Egypt, Cairo 11837, Egypt; 6 Department of Biochemistry, Faculty of Medicine, Minia University, Minia 61519, Egypt; 7 Department of Pharmacognosy, Faculty of Pharmacy, ALSalam University, Kafr El Zayat 31616, Egypt; fatma.mokhtar@sue.edu.eg; 8 Deanship of Scientific Research, Umm Al-Qura University, Makkah 21955, Saudi Arabia; hforaby@uqu.edu.sa; 9 Department of Crop Science, Faculty of Agriculture, Zagazig University, Zagazig 44519, Egypt; 10 Science and Technology Unit (STU), Umm Al-Qura University, Makkah 21955, Saudi Arabia; amesmail@uqu.edu.sa; 11 Department of Pharmacognosy, Faculty of Pharmacy, Minia University, Minia 61519, Egypt

**Keywords:** *Psidium guajava*, metabolic profiling, gastro protective, docking

## Abstract

One of the most severe human health problems is gastric ulceration. The main aim of our study is to explore the gastroprotective effect of the *Psidium guajava* seeds extract (PGE). Metabolic profiling based on LC-HRMS for the extract led to the dereplication of 23 compounds (**1**–**23**). We carried out a gastric ulcer model induced by indomethacin in male albino rats in vivo and the extract of PGE was investigated at a dose of 300 mg/kg in comparison to cimetidine (100 mg/kg). Furthermore, the assessment of gastric mucosal lesions and histopathology investigation of gastric tissue was done. It has been proved that *Psidium guajava* seeds significantly decreased the ulcer index and protected the mucosa from lesions. The antiulcer effect of *Psidium guajava* seed extract, which has the power of reducing the ensuing inflammatory reactions, can counteract the inflammation induced by indomethacin by the downregulation of relative genes expression (*IL-1β*, *IL-6*, and *TNF-α*). Moreover, PGE significantly downregulated the increased *COX-2*, *TGF-β*, and *IGF-1* relative genes expression, confirming its beneficial effect in ulcer healing. Moreover, the possible PGE antioxidant potential was determined by in vitro assays using hydrogen peroxide and superoxide radical scavenging and revealed high antioxidant potential. Additionally, on the putatively annotated metabolites, an in silico study was conducted, which emphasized the extract’s antiulcer properties might be attributed to several sterols such as stigmasterol and campesterol. The present study provided evidence of *Psidium guajava* seeds considered as a potential natural gastroprotective agent.

## 1. Introduction

*Psidium guajava* (Family: *Myrtaceae*) is a tropical edible fruiting plant. They are evergreen trees indigenous to subtropical and tropical climatic regions, it originated from Latin America and was cultivated in many locations of Asia, Africa, and Europe [1]. The plant is an economically important crop cultivated for fruit production such as food and raw material in food industry [2,3]. Moreover, *P. guajava* has a wide range of medicinal applications [4]. *P. guajava* is a plentiful source of diverse secondary metabolites including phenolic acids (chlorogenic, ferulic and caffeic acids) [5], flavonoids (quercetin, kaempferol, and morin glycosides) [6], tannins (guavins A, B, C, D) [7], ellagitannins (casuarinin, flavonotannins; psidinins A, B, C) [8], Benzophenone glycosides (guavinoside B) [9,10]. Guava leaves and bark were assigned to treat gastrointestinal disorders; particularly diarrhea [11]. In the last decades, several studies were performed using the decocted or infused extracts of leaves, fruits, stems, and flower buds to study their different biological activities [12,13,14,15,16,17,18,19]. The anti-inflammatory, antihypertensive, blood glucose lowering effect, antiviral and cough sedative activities are the key factors which labelled the guava fruit to be a perfect candidate to protect against COVID-19 infection especially for chronic diseased individuals [20]. Guava seeds (PGE) as a waste product of the commercially important fruit was evaluated as an inhibitor for the proliferation of the human erythroleukemic cells with low toxicity [21], the seed oil contains unsaturated fatty acids especially, linoleic acid and possessed a high antioxidant activity [22]. In addition to seed fatty acids, the seeds were found to be rich with proteopolysaccharides with potent anti-inflammatory and immunomodulatory effects [23], peptides from guava seeds were reported to perform antimicrobial effects against gram-negative bacteria [24], the phenolic content of the seeds extracted by supercritical fluid extraction performed a high antioxidant activity [25]. The anticorrosion potential of *Psidium guajava* seeds on carbon steel in an acid medium was reported as an ecofriendly application [26]. Guava seeds are considered a rich reservoir of variable potent phytochemical constitutes, so we investigated for the first time the gastroprotective and antioxidant of the *Psidium Guajava* seeds as well as their anti-inflammatory potential. Moreover, we carried out LC-MS analysis to reveal the chemical entities of *Psidium guajava* seeds. Finally, a docking study was carried within three different active sites, which includes the gastric proton pump, muscarinic receptors as well as H2 receptors.

## 2. Materials and Methods

### 2.1. Plants Material

The seeds of *Psidium guajava* were collected in September 2021 from Minia area, Egypt. Mr. Abdallah Salem authenticated the plant (at Minia University, Minia, Egypt). A voucher specimen (PS 2-2021) was conserved at Deraya University.

### 2.2. Extraction of Psidium guajava Seeds

One kilogram of dried *Psidium guajava* seeds were extracted three times by maceration in methanol at an ambient temperature. The alcoholic extract was vacuum-dried to produce a dense residue (100 g).

### 2.3. Metabolic Profiling

LCMS was performed by using a Synapt G2 HDMS quadrupole time-of-flight hybrid mass spectrometer (Waters, Milford, CT, USA). One mg of the sample was injected into the BEH C18 column, at 40 °C, and connected to the guard column. A gradient elution was used, starting from no solvent A and 0.1% formic acid in water to 100% acetonitrile as solvent B. MZmine 2.12 San Diego, USA was used to investigate MS data using mzML format converted with ProteoWizard Palo Alto, CA, USA [27].

### 2.4. Docking Study

Investigated compounds 3D structures were downloaded from PubChem and prepared as reported earlier [28]. Compounds were docked against a gastric proton pump (PDB 5YLU), M3 muscarinic receptor (PDB 5ZHP) obtained from http://www.rcsb.org/ (accessed on 23 March 2022) and against a H2 receptor as obtained from GPCR data base. Docking was done in the active sites of target protein as recognized by co-crystalized ligands. Docking was done using Autodock Vina [29] using cubic grid box with 25 Å side and exhaustiveness of 16. Similarly docking was also done using the same procedure against other targets involved in inflammation including COX-1 (PDB 1EQH), COX-2 (PDB 3LN1), TNF-α (PDB 2AZ5), TGF-β (PDB 5E8S), EGFR (PDB 1M17) and IGFR (PDB 5XFS).

### 2.5. Animal Model

The experiment was conducted on 150–200 g Albino rats (Wistar) kept under normal conditions (room temperature 24–27 °C, moisture 60–65%) with a 12-h light-dark cycle. Rats were fasted for 24 h before the test to guarantee an empty stomach. The animals were housed in standard cages with free access to water and food. The animals were acclimatized for seven days prior to the experiment start day, and all circumstances were designed to eliminate animal suffering. To eliminate differences owing to diurnal rhythms as possible regulators of stomach functions, all rats were used in the experiment at the same time of day [30,31]. The rats were separated into four groups: A, B, C and D, each of which had 6 rats. The normal control group, Group A received only the vehicle (0.5 percent CMC solution). Group B (positive control) was given the vehicle (0.5 percent CMC solution). Group C (market comparable reference group) was administered oral cimetidine (100 mg/kg p.o) (oral administration), whereas Group D was given guava seed extracts of 300 mg/kg p.o. (dissolved in a 0.5% CMC solution). After 1 h, groups B, C, and D received a single acute dosage of indomethacin (IND) (40 mg/kg) orally to induce gastric ulcers. All animals of the four groups were killed four hours after starting the experiment [32,33]. The stomach was cut and the larger curvature of the stomach was sliced apart. The stomachs were placed flat on a cork board after being washed under a steady stream of water. The ulcer index was obtained by counting the number of ulcer spots in the glandular region of the stomach in both control and drug-treated animals [30,34].

### 2.6. Gastric Mucosal Lesions Evaluation

The ulcer index (UI) was determined using a 0–3 scoring system and an inspection of the stomachs using an eyepiece. The severity factor was determined as follows: level 0 = no lesions, level 1 = lesions, level 2 = lesions, level 3 = lesions. Lesions with a length of less than 1 mm were classified as level 1, lesions with a length of 2–4 mm were classified as level 2, and lesions with a length of more than 4 mm were classified as level 3. Each rat’s lesion score was determined by multiplying the number of lesions by their respective factor. The drug’s preventative index (PI) was determined by the use the following formula:

PI = (UI of indomethacin group − treated group) × 100\(UI of indomethacin group) [35].

### 2.7. Histopathological Examination

The stomach tissue was preserved in a 10% ethanol formalin solution and treated with graded ethanol, paraffin wax, and xylene before being cut into slices using a microtome. The slices were viewed under a microscope after being stained with Eosin stain and Hematoxylin. The various histological indicators were examined [36].

### 2.8. Quantitative Real-Time Polymerase Chain Reaction (qRT-PCR)

Quantitative Real-Time Polymerase Chain Reaction is discussed in detail in the Supplementary Materials, as mentioned in Table S1.

### 2.9. In Vitro Antioxidant Activity

The H_2_O_2_ and SOD scavenging activities of *Psidium Guajava* seed crude extract are thoroughly discussed in the Supplementary Materials.

### 2.10. Statistical Analysis

All of the data were set as a mean ± SD. A one-way analysis of variance (ANOVA) was used to evaluate the data of all groups, followed by the Dunnett’s test with statistical significance at *p* < 0.05.

## 3. Results and Discussion

**Figure 1:**
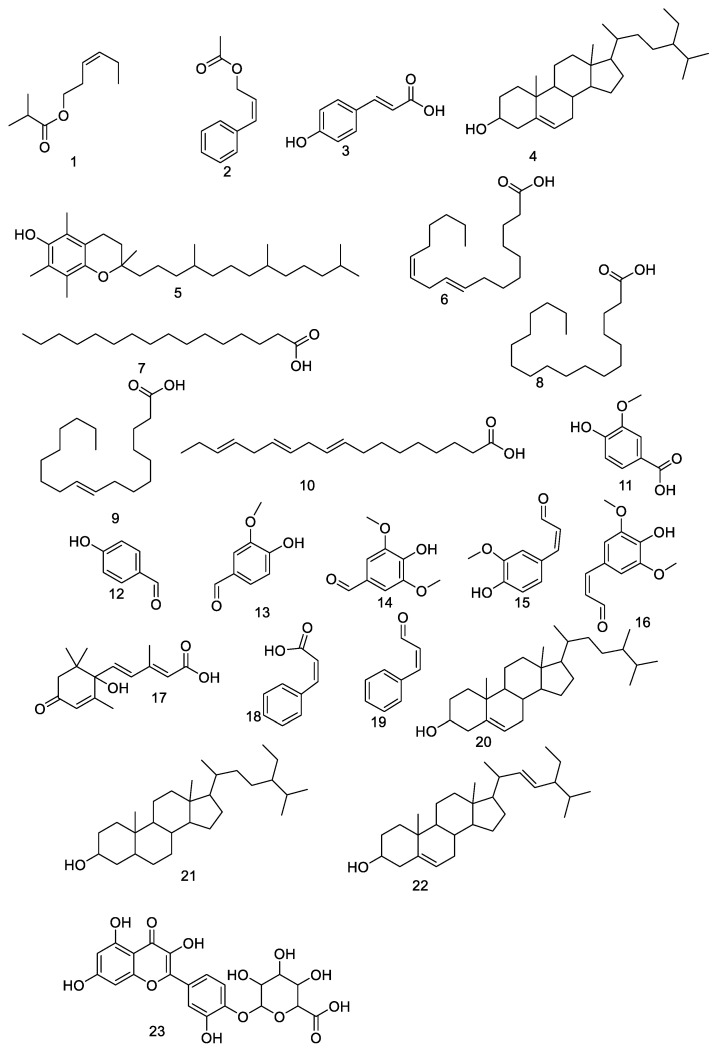
Putative compounds identified from *Psidium guajava* seed.

**Figure 2:**
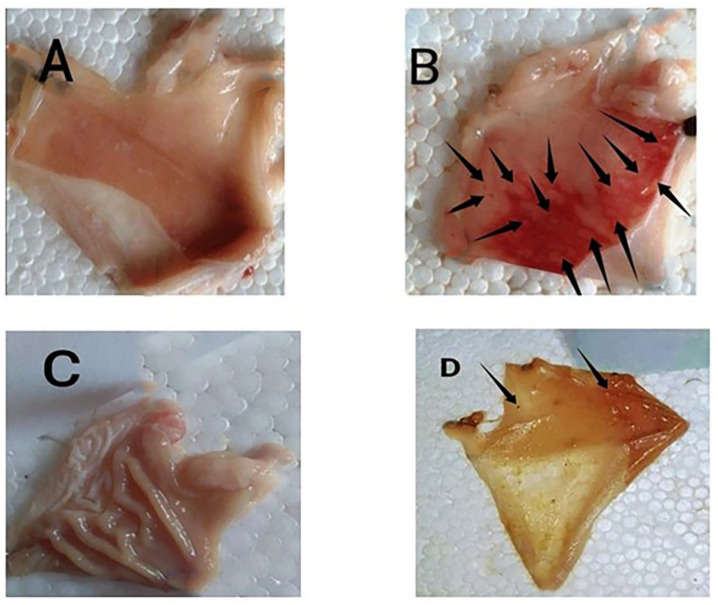
(**A**–**D**) Photos of rat stomachs. (**A**) (normal group), (**B**) (positive control group), (**C**) (cimetidine group), (**D**) (guava seed extract group). Effect of Guava seed extract on the gastric lesion severity (gross examination). (**A**) Control: intact gastric mucosa tissues; (**B**) indomethacin (ulcer): severe lesions are seen with extensive visible hemorrhagic necrosis of gastric mucosa; (**C**) cimetidine-treated rats: mild lesions of the gastric mucosa are observed compared to the lesions in indomethacin-induced ulcer rats; (**D**) guava seed extract-treated rats: nearly normal gastric mucosa tissues.

Metabolic profiling using HR-LCMS was performed on the crude extract of *Psidium guajava* seeds to investigate the chemical constituents that might be responsible for the protective role against ulcer formation. The identified compounds as shown in Figure 1 were assorted into different chemical classes. Identification of the compounds was achieved based on HR-ESIMS data and comparison with literature data as follows: Cis-3-Hexenyl isobutyrate (**1**) [37], cinnamyl acetate (**2**) [38], coumaric acid (**3**) [39], β-sitosterol (**4)** [39], α-tocopherol (**5**) [39], linoleic acid (**6**) [39], palmitic acid (**7**) [39], stearic acid (**8**) [39], oleic acid (**9**) [39], linolenic acid (**10**) [39], vanillic acid (**11**) [39], 4-Hydroxybenzaldehyde (**12**) [39], vanillin (**13**) [39], syringaldehyde (**14**) [39], coniferylaldehyde (**15)** [39], sinapaldehyde (**16**) [39], abscisic acid (**17**) [39], cinnamic acid (**18**) [39], cinnamaldehyde (**19**) [39], campesterol (**20**) [39], stigmastanol (**21**) [39], stigmasterol (**22**) [39] and quercetin 4′-glucuronide (**23**) [40]. Guava seed extract provided substantial protection against the ulcerogenic effects of indomethacin in a dose-dependent manner (Table 1). We carried out the study as follows: group A was a normal group that didn’t have any ulcers, while group B (indomethacin) showed lesions that were hemorrhagic and shaped in a linear or dotted pattern. Furthermore, group C (cimetidine) showed mild lesions. Finally, group D (guava seed extract) had a similar number of ulcers to the normal group (Figure 2). Our results showed that the indomethacin group (group B) had a lesion index of (117.32 ± 23.3), while the cimetidine treated group (group C) had a lesion index of (2.0 ± 1.01) and preventive index of 98.2%. On the other hand, group D (IND+ *Psidium Guajava* seeds extract) had the lowest ulcer index (1.33 ± 0.33) and a high preventive index (98.6%). These results proved the powerful gastroprotective potential of guava seed extract, which showed a higher preventive index (98.6%) comparable to the cimetidine group. Moreover, our extract exerts a powerful gastroprotective effect, more than many natural extracts such as *Ocimum forskolei,* which has a preventive index 91.17% [41], and *Elaeocarpus grandis,* which has a preventive index of 92.9% [42].

### 3.1. Histopathological Studies

**Figure 3:**
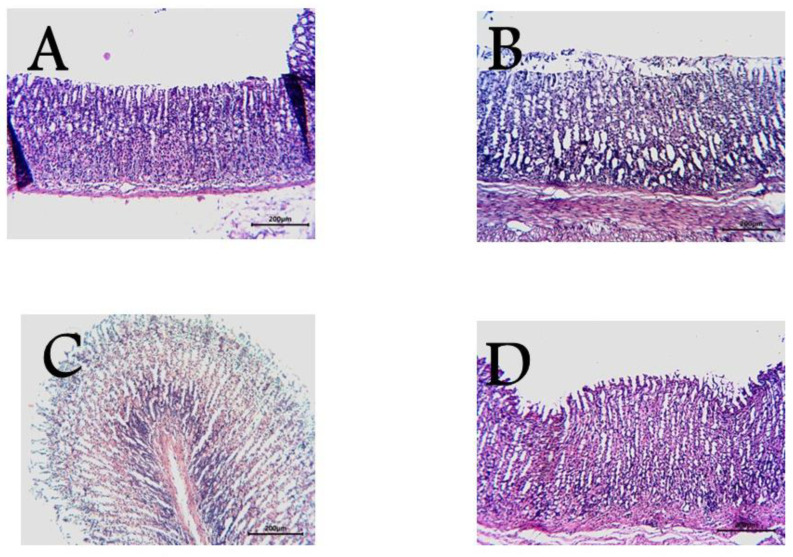
Light micrographs showing the effect of guava seed extract on Indomethacin-induced gastric ulcers of rats. (**A**) Group A (normal mucosa), (**B**) Group B (Indomethacin-induced gastric mucosal lesions), (**C**) Group C (cimetidine treated group show low mucosal lesions), (**D**) Group D (pretreatment of rats with guava seed extract) show no mucosal alterations.

Moreover, the pharmacological and biochemical findings are compatible with histological investigations of stomach tissue. The normal stomach mucosa is shown in Figure 3A. The negative control group (Figure 3B) showed ulceration and bleeding on the mucosa. The positive control group (Figure 3C) had cimetidine. Guava seed extract pretreatment of rats resulted in a lack of mucosal ulcers and bleeding, as shown in Figure 3D.

### 3.2. Docking Study

**Figure 4:**
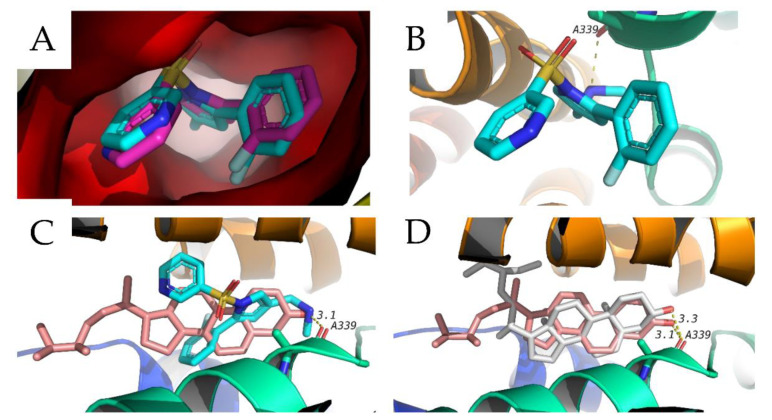
Docking of identified compounds against gastric proton pump (PDB 5YLU). (**A**) Validation of docking procedure with target protein. (**B**) Interactions of vonoperazone (blue) with the active site of protein. (**C**) Docking pose of campesterol (pink) overlapped with co-crystalized ligand (blue). (**D**) Docking pose of stigmasterol (white) and campesterol (pink).

In this study, we used a molecular docking technique to predict potential mechanisms of action of the identified compounds acting as an antiulcer. Twenty-three compounds were docked against three antiulcer targets including the gastric proton pump, muscarinic receptor and H2 receptor. Two of these targets are available on the protein date bank, which are the proton pump and muscarinic receptor under 5YLU and 5ZHP, respectively. The structure of the H2 receptor was obtained in homology model form in the GPCR data base. Validation of docking procedure was done by redocking of the co-crystalized ligand and calculation of RMSD between docked and crystalized poses. An RMSD of less than 2 Å is considered acceptable (Figure 4A shows and example of the validation of the gastric proton pump). Docking results are shown in Table 2, and show that several sterol compounds have docking scores better than the co-crystalized ligand vonoprazan in cases with the gastric proton pump. These compounds include β-Sitosterol, campesterol, stigmastanol and stigmasterol. The scores of these compounds with gastric proton pump is better than their scores with the other two targets. In addition to these sterols, Quercetin-4′-glucuronide showed a docking score with the same target, the gastric proton pump, that is very close to the co-crystalized ligand. The docked sterols were able to maintain the same hydrogen bond that is formed by vonoprazone with A339 of the gastric proton pump (Figure 4B). Among those sterols, campesterol showed the best docking score (−9.9 kcal/mol) with proton pump protein. Its docking pose overlapped with the docking position of vonoperazone, forming the same hydrogen bonds as well as hydrophobic interactions, as shown in Figure 4C. Another example is the next sterol, stigmasterol (−9.7 kcal/mol), that showed a similar docking pose to campesterol, as shown in Figure 4D. These results suggest that these sterols might be responsible for the antiulcer effect through the inhibition of the gastric proton pump, which might require further follow-up studies to investigate this effect. In addition, the same twenty-three identified compounds were also docked in the active sites of several inflammatory targets and mediators which include COX-1, COX-2, TNF-α, TGF-β, EGFR, and IGFR. This was conducted to investigate whether the antiulcer effect of these compounds could be mediated through the modification of any of these inflammatory mediators. The results of this docking study are also shown in Table 2. Several sterols were able to hit some of these inflammatory targets; for example, stigmasterol had good docking scores with TGF-β (−9.6 kcal/mol) and EGFR (−9.4 kcal/mol). Another example of a nonsteroidal compound that showed good results with one of these inflammatory mediators is quercetin-4′-glucuronide, which showed its best docking score with TGF-β (−9.5 kcal/mol). These results suggest that other mechanisms could also be involved in the antiulcer effect of the study extracts and open the door for further investigation in this important area.

### 3.3. Effect of Psidium guajava Seed Extract on Expression of TNF-α, IL-1β, IL-6, TGF-β, COX-2, and IGF-1

After mucosal damage, the healing of a stomach ulcer is a complex physiological process including tissue auto-repair, reperfusion, and regeneration. A stomach ulcer is a lesion comprised of connective and granular tissues with fibroblasts and macrophages, as well as the generation of proliferating microvessels at the ulcer base and ulcer edge via angiogenesis. This lesion is surrounded by non-necrotic mucosa, which creates the ulcer edge that heals too quickly, resulting in the formation of an ulcer scar. There are four stages to the ulcer healing process: Indomethacin-induced inflammation, ulcer formation as evidenced by inflammatory cell infiltration, tissue necrosis, and lastly, the creation of granulation tissue and ulcer margin [43].

**Figure 5:**
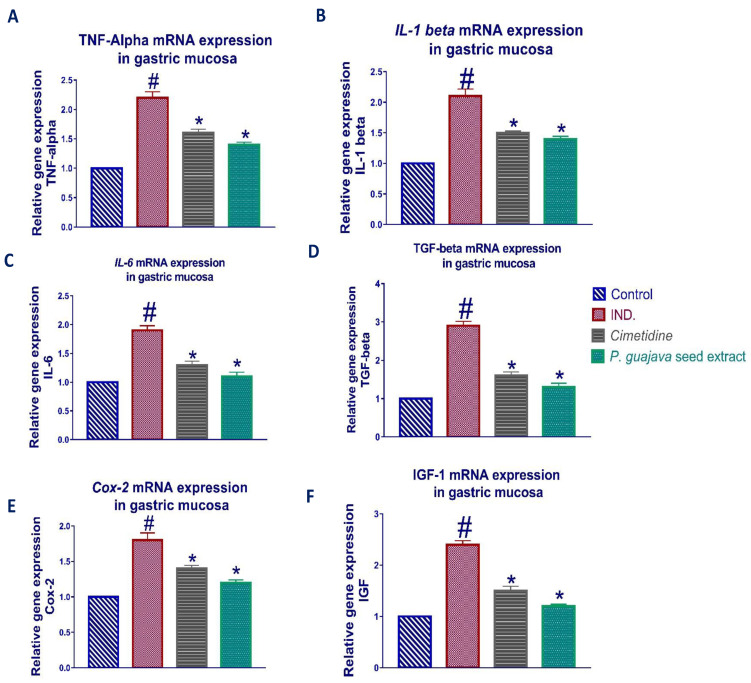
*TNF-α* (**A**), *IL-1β* (**B**), *IL-6* (**C**), *TGF-β* (**D**), *COX-2* (**E**), and *IGF-1* (**F**) relative gene expression in gastric mucosa tissue of different groups of rats was assessed using quantitative RT-PCR. After normalization to *GAPDH*, the data reflect a fold difference in expression compared to the normal control group. The bars show the mean ± standard deviation. To see if there is a significant difference between groups, a one-way ANOVA test is performed, with # *p* < 0.05 compared to the normal control group and * *p* < 0.05 compared to the indomethacin induced group.

Upon activation of immune cells, a variety of proinflammatory cytokines such as interleukin-1beta (*IL-1β*), interleukin-6 (*IL-6*), and tumor necrosis factor-α (*TNF-α*) are secreted from macrophages. A proinflammatory cytokine, *IL1β,* regulates a variety of genes involved in the inflammatory reaction and related tissue damage, such as compromising the function of entero-chromaffin-like cells. In addition, *IL-1β* inhibits the proliferation of gastric epithelial cells [44], an effect that involves tyrosine kinase, protein kinase C, and multiple mitogen-activated protein kinases. The inflammation triggered by *IL-1β*, *Il-6* and *TNF-α* can be antagonized by the antiulcer action of *Psidium guajava* seed extract, which attains the capability of suppressing the resultant inflammatory responses, as shown in Figure 5.

The induction of *COX-2* in fibroblasts, epithelial, parietal, and mononuclear cells of the mucosa, and in response to *IL-1β* elevation, is related with indomethacin-induced gastritis. The results showed a marked reduction of the elevated *COX-2* relative gene expression upon treatment with *Psidium guajava* seed extract, which further confirms its protective role in ulcer healing, as shown in Figure 5.

*IL-1β* contributes to the initial upregulation of *TGF-β*, and *IGF-1* in the base of indomethacin-evoked ulcers in a rat stomach [45]. Although *TGF-β* inhibits cell proliferation in a variety of cells, it induces angiogenesis, cell migration, and increases extracellular matrix (ECM) production, all of which are required for gastrointestinal ulcer healing at the intermediate and late stages of the healing process. It also acts as an anti-inflammatory cytokine [46]. Macrophages are activated following indomethacin-induced inflammation, which increases the release of active *TGF-β* with increasing plasmin activation [47].

Furthermore, *IGF-1* expression can contribute to the acceleration of ulcer healing in both the early and late phases of the healing process [46]. *IGF-1*, a major mediator of soft tissue regeneration, is released from platelet granules shortly after damage and plays a crucial role in angiogenesis and epithelialization in gastric ulceration [48]. Accordingly, we found that the *IGF-1* mRNA level was significantly increased in the stomach mucosa following ulceration in normal rats. Finally, after treatment with *Psidium guajava* seed extract, the results showed marked downregulation of the elevated *TGF-β*, and *IGF-1* relative gene expression, as shown in Figure 5.

### 3.4. In Vitro Antioxidant Activity of Psidium guajava Seed Extract

#### 3.4.1. Hydrogen Peroxide Scavenging Activity

**Figure 6:**
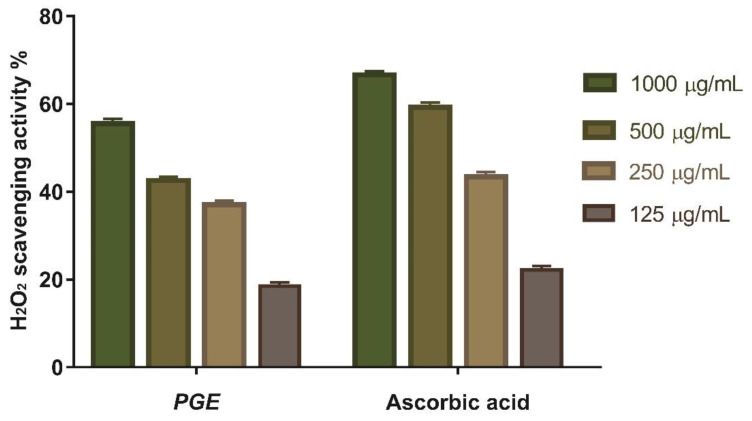
H_2_O_2_ radical scavenging activity of PGE at different concentration (1000 µg/mL, 500 µg/mL, 250 µg/mL, and 125 µg/mL). Bars represent mean ± standard deviation (SD). Significant difference between groups is analyzed by a two-way ANOVA test.

This study looked at the antioxidant activity of *Psidium Guajava* seed extract as a scavenger potential against (H_2_O_2_). According to the findings, the maximum H_2_O_2_ radical scavenging activity of PGE was 55.55% at a 1000 µg/mL concentration. When compared to conventional ascorbic acid (IC_50_ = 182.7 µg/mL), *Psidium guajava* seed extract suppressed the formation of H_2_O_2_ in a dose-dependent manner (Figure 6).

#### 3.4.2. Superoxide Radical Scavenging Activity

**Figure 7:**
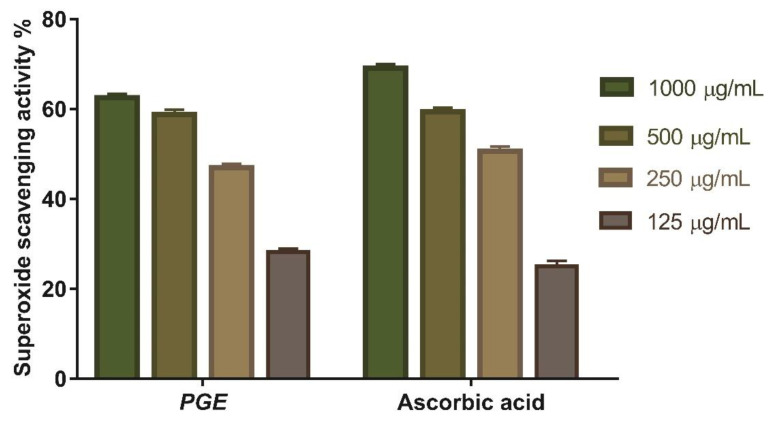
Superoxide radical scavenging activity of *Psidium guajava* seed extract at different concentration (1000 µg/mL, 500 µg/mL, 250 µg/mL, and 125 µg/mL). Bars represent mean ± SD (standard deviation). Significant difference between groups is analyzed by a two-way ANOVA test.

The superoxide scavenging (SOD) activity of *Psidium guajava* seed extract was determined. The scavenging potential of PGE and the standard increase are concentration dependent (Figure 7), with *Psidium guajava* seed extract showing the maximum superoxide radical scavenging activity. *Psidium guajava* seed extract had 62.5% superoxide scavenging efficacy at a 1000 μg/mL concentration. The concentration of *Psidium guajava* seed extract needed for 50% inhibition (IC_50_) was found to be 147.1 µg/mL compared to (IC_50_) 158.8 μg/mL for ascorbic acid.

## 4. Conclusions

The present research examined the possible gastroprotective effect of *Psidium Guajava* seed extract against NSAID-induced gastric ulcers, as well as its antioxidant activity (hydrogen peroxide scavenging activity and superoxide radical scavenging activity). Metabolomic profiling via LC-HRMS-based chemical profiling of *Psidium guajava* revealed the abundance of many putative active compounds. *Psidium guajava* seed extracts significantly decreased the ulcer index and protected the mucosa from lesions. The antiulcer effect of *Psidium guajava* seed extract downregulated the cytokines expression (*IL-1β*, *IL-6*, and *TNF-α*). Moreover, PGE significantly downregulated the increased *COX-2*, *TGF-β*, and *IGF-1* relative gene expressions, confirming its beneficial effect in ulcer healing. Moreover, the identified compounds were also docked in the active sites of several inflammatory targets and mediators, which include COX-1, COX-2, TNF-α, TGF-β, EGFR, and IGFR. The gastroprotective effect could be potentially attributed to the effect of several sterols such as stigmasterol and campesterol, as suggested by docking studies. Our experiment proved the possible use of *Psidium guajava* seeds as a protective agent against ulceration, and a possible alternative to antiulcer agents. These results might need further investigation to support this potential effect, but they highlight the mechanism of the gastro protective and antioxidant effect of *Psidium guajava* seeds.

## Figures and Tables

**Table 1:** Antiulcer activity of guava seed extract.

Groups	Level 1	Level 2	Level 3	Ulcer Index	PI%
Group A	-	-	-	-	-
Group B	22 ± 3.02	26.66 ± 2.17	14 ± 5.26	117.32 ± 23.3	-
Group C	1.31 ± 0.32	0.3 ± 0.31	0	2.0 ± 1.01 ***	98.2%
Group D	1.33 ± 0.57	0	0	1.33 ± 0.33 ***	98.6%

Data are represented as mean ± SD. One-way ANOVA followed by Dunnett’s multiple comparison tests software (accessed on 15 April 2022) were used for statistical analysis. Results were regarded significant as follows: *** *p* < 0.001.

**Table 2:** Docking results of identified compounds against antiulcer targets.

	Docking Score (Kcal/Mol)								
	Anti-Ulcer Targets			Anti-Inflammatory Targets					
Ligand	Proton Pump (5YLU)	M3 Receptor (5ZHP)	H2-Receptor Model	COX-1 (1EQH)	COX-2 (3LN1)	TNF-α (2AZ5)	TGF-β (5E8S)	EGFR (1M17)	IGFR (5XFS)
cis-3-Hexenyl-isobutyrate	−5.6	−6.0	−5.8	−6.1	−6.0	−5.1	−5.4	−5.2	−4.7
Cinnamyl-acetate	−6.3	−6.9	−6.6	−6.9	−7.2	−5.7	−6.5	-6.1	−5.8
Coumaric-acid	−6.3	−6.7	−5.8	−6.3	−6.9	−5.7	−6.2	−5.7	−6.1
β-SITOSTEROL	−9.6	−8.9	−5.8	−7.7	−7.0	−8.5	−8.7	−8.5	−8.1
α-Tocopherol	−8.4	−9.2	−6.8	−8.7	−8.2	−7.5	−8.0	−7.6	−7.3
linoleic acid	−6.5	−7.0	−6.8	−6.7	−7.3	−5.9	−6.0	−5.3	−5.6
Palmitic acid	−6.1	−6.8	−6.4	−6.2	−7.0	−5.2	−5.7	−5.0	−5.2
Stearic acid	−6.1	−6.4	−6.4	−6.2	−7.0	−5.0	−5.8	−5.2	−5.2
Oleic acid	−6.1	−7.1	−6.5	−7.2	−7.2	−5.7	−5.8	−5.6	−5.5
Linolenic acid	−6.6	−7.2	−7.1	−6.8	−7.5	−5.7	−6.6	−5.7	−5.8
Vanillic acid	−6.0	−6.3	−5.6	−6.3	−6.3	−5.4	−6.6	−6.2	−5.5
p-Hydroxybenzaldehyde	−5.1	−5.8	−5.0	−5.9	−5.8	−4.9	−5.8	−5.5	−4.9
Vanillin	−5.4	−5.9	−5.4	−6.0	−5.8	−5.3	−6.2	−5.5	−5.2
Syringaldehyde	−5.4	−6.0	−5.6	−6.1	−6.0	−5.2	−5.3	−5.2	−5.2
Coniferylaldehyde	−6.1	−6.6	−5.5	−6.6	−6.8	−5.6	−6.0	−5.6	−6.0
Sinapaldehyde	−6.0	−6.3	−5.6	−5.8	−6.7	−5.4	−6.2	−5.9	−5.8
Abscisic acid	−7.9	−8.6	−6.8	−8.0	−8.1	−7.3	−7.0	−6.8	−6.8
Cinnamic acid	−6.3	−6.7	−6.1	−6.2	−6.9	−5.9	−6.0	−5.6	−5.9
Cinnamaldehyde	−5.6	−6.0	−5.5	−6.0	−6.1	−5.6	−5.4	−5.0	−5.3
Campesterol	−9.9	−8.7	−5.8	−7.6	−6.9	−8.8	−8.9	−9.1	−8.3
Stigmastanol	−9.6	−8.5	−6.2	−7.5	−7.4	−8.6	−8.8	−8.3	−8
Stigmasterol	−9.7	−8.5	−6.3	−7.9	−7.5	−8.9	−9.6	−9.4	−8.5
Quercetin-4′-glucuronide	−9.3	−8.6	−7.8	−7.6	−9.0	−8.3	−9.5	−8.5	−8.4
Co-crystalized Ligand	−9.5	−11.2	-	−9.6	−13.2	−9.2	-	−7.1	−10.0

## Data Availability

Data is contained within the article and supplementary material.

## Supplementary Materials

The following supporting information can be downloaded at: https://www.mdpi.com/article/10.3390/antiox11071230/s1, Table S1: Primer sequence of the genes included in the study [35,49,50,51,52].
