# Supplementary material for: Antiulcer Potential of Psidium guajava Seed Extract Supported by Metabolic Profiling and Molecular Docking
Source: Antioxidants (Basel). 2022 Jun 23;11(7):1230. doi: 10.3390/antiox11071230 (PMC9311959; doi:10.3390/antiox11071230)
Supplement: Supplementary file 1 [file antioxidants-11-01230-s001.zip › antioxidants-1753252-supplementary.pdf]

Antiulcer Potential of *Psidium guajava* Seed Extract Supported by Metabolic Profiling and Molecular Docking

Nourhan Hisham Shady [1], Hend Samy Abdullah [2], Sherif A. Maher [3], Amgad Albohy [4,5], Mahmoud A. Elrehany [3,6], Fatma Alzahraa Mokhtar [7], Hesham Farouk Oraby [8,9], Ahmed M. Shawky [10] and Usama Ramadan Abdelmohsen [1,11,*]

[1] Department of Pharmacognosy, Faculty of Pharmacy, Deraya University, Universities Zone,
New Minia City 61111, Egypt; norhan.shady@deraya.edu.eg
[2] Faculty of Pharmacy, Deraya University, Universities Zone, New Minia City 61111, Egypt; hend.samy_1180443@student.deraya.edu.eg
[3] Department of Biochemistry, Faculty of Pharmacy, Deraya University, Universities Zone,
New Minia City 61111, Egypt; sherif.ali@deraya.edu.eg (S.A.M.);
sherif.ali@deraya.edu.eg mahmoud.elrehany@deraya.edu.eg
[4] Department of Pharmaceutical Chemistry, Faculty of Pharmacy, The British University in Egypt,
El-Sherouk City, Cairo 11837, Egypt; amgad.albohy@bue.edu.eg
[5] The Center for Drug Research and Development (CDRD), Faculty of Pharmacy, The British University in Egypt, El-Sherouk City, Cairo 11837, Egypt
[6] Department of Biochemistry, Faculty of Medicine, Minia University, Minia 61519, Egypt
[7] Department of Pharmacognosy, Faculty of Pharmacy, ALSalam University, Kafr El Zayat 31616, Al Gharbiya, Egypt; fatma.mokhtar@sue.edu.eg
[8] Deanship of Scientific Research, Umm Al-Qura University, Makkah 21955, Saudi Arabia; hforaby@uqu.edu.sa
[9] Department of Crop Science, Faculty of Agriculture, Zagazig University, Zagazig 44519, Egypt
[10] Science and Technology Unit (STU), Umm Al-Qura University, Makkah 21955, Saudi Arabia;
amesmail@uqu.edu.sa
[11] Department of Pharmacognosy, Faculty of Pharmacy, Minia University, Minia 61519, Egypt
* Correspondence: usama.ramadan@mu.edu.eg

Materials and Methods

Quantitative Real-Time Polymerase Chain Reaction (qRT-PCR)

Approximately 50 mg of the gastric mucosa tissue was homogenized by ultrasonic homogenizer (SFX 550 Branson Digital Sonifier® ultrasonic cell disruptor/homogenizer is versatile. Danbury, CT, USA) in 0.5 mL of TRIzol TM reagent (Amresco, Solon, OH, USA). RNA was extracted from gastric mucosa tissue using the TRIzol TM RNA

Extraction Reagent (Amresco, Solon, OH, USA) as instructed by the manufacturer. The overall RNA concentration was estimated at A260 nm, and the purity was measured based on the ratio A260/A280. Samples with purity ≥ 1.7 were used for qRT-PCR. GAPDH was used as a reference housekeeping gene. cDNA synthesis was performed for equivalent amounts of total RNA in all samples using the RevertAid H Minus First Strand cDNA Synthesis Kit (#K1632, Thermo Science Fermentas, St. Leon-Ro, Germany) as directed by the manufacturer. Real-time PCR was conducted with single-stranded cDNAs. The sequences of the used primers are shown in (Table S1) PCR reactions were conducted by SYBER Green (#K0251, Thermo Scientific Fermentas St. Leon-Ro, Germany-Maxima SYBER Green qPCR Master Mix (2X)) using a StepOne Real-Time PCR Detection System (Applied Biosystems).

Table S1: Primer sequence of the genes included in the study

Primer	Sequence 5' to 3'
TNF-α	Forward CAG AGG GAA GAG TTC CCC AG<br>Reverse CCT TGG TCT GGT AGG AGA CG
IL-1β	Forward GAG GCT GAC AGA CCC CAA AAG AT<br>Reverse GCA CGA GGC ATT TTT GTT GTT CA
IL-6	Forward CGC CCT AGT GGG GTA GTA GT<br>Reverse CAG TCA GAC CCT TCA CCG TC
TGF-β	Forward TTG CCC TCT ACA ACC AAC ACA A<br>Reverse GCT TGC GAC CCA CGT AGT A
COX-2	Forward CAT TGA CCA GAG CAG AGA GAT<br>Reverse TTC TTG AAT GTC CTC TCT TTC
IGF-1	Forward CCT CAT TAT CCC TGC CCA CCA A<br>Reverse GCT GGT GAA GGT GAG CAA GC

GAPDH	Forward ACC AAC TGC TTA GCC CCC C<br>Reverse GCA TGT CAG ATC CAC AAC GG

Real-time polymerase chain reaction (qRT-PCR) achieved using 20 µl of RealMOD Green qRT-PCR Mix kit (iNtRON biotechnology) with 0.02 µg RNA per reaction and 10 Pmol of unique primers, for 30 cycles of 95 ˚C for 10 s. and 60 ˚C for 1 min. The comparative Ct (threshold cycle) approach was used to assess the relative concentrations of the products. The relative expression was determined using formula $2^{(-\Delta\Delta Ct)}$. They were scaled compared to the controls.

In Vitro Antioxidant Activity

Hydrogen Peroxide Scavenging Activity

The reaction with a defined amount of exogenously provided H_2O_2 was used to determine the hydrogen peroxide (H_2O_2) scavenging activity that reflects the anti-oxidative capacity of *Psidium Guajava* seed extract. Colorimetric analysis was used to estimate the residual H_2O_2 [1,2] [3]. In brief, 20µl of the extract mixed with 500 µl of H_2O_2 and incubated at 37ºC for 10 minutes. After that, 500 µl of enzyme/3, 5-dichloro-2-hydroxyl-benzenesulfonate solution added and incubated at 37ºC for 5 minutes. Colorimetrically, the intensity of the colored product measured at 510 nm. Positive control was ascorbic acid. The percentage of H_2O_2 scavenging activity was determined by comparing the results of the test with those of the control using the following formula:

$$\text{scavenging activity} = \frac{\text{A control} - \text{A sample}}{\text{A control}} \times 100$$

IC_{50} of each sample calculated after performing the assay at four different concentrations (1000 µg/mL, 500 µg/mL, 250 µg/mL, and 125 µg/mL) using Graph pad prism 7 software.

Superoxide Radical Scavenging Activity

The superoxide anion scavenging activity was measured as described by Sreenivasan et al. [4,5]. The superoxide anion radicals were formed in a Tris – HCl buffer (16 mM, pH8.0) containing 90 µl of NBT (0.3 mM), 90 µL of NADH (0.936 mM), 0.1mL of *Psidium Guajava* seed extract (125, 250, 500, and 1000 g/mL), and 0.8 mL Tris – HCl buffer (16 mM, PH 8.0). The reaction initiated by adding 0.1 mL Phenazine Methosulfate (PMS) solution (0.12 mM) to the mixture, which was then incubated at 25˚C for 5 minutes, and at 560 nm, the absorbance was measured. Ascorbic acid was selected as a reference. The percentage inhibition was obtained by comparing the test findings to those of the control using the formula below:

$$\text{Superoxide scavenging activity} = \frac{\text{A control} - \text{A sample}}{\text{A control}} \times 100$$

IC_{50} was calculated using Graph pad prism 7 software by performing the test at four different concentrations.

Reference

1. Hassan, H.; Abdel-Aziz, A.J.F.; toxicology, c. Evaluation of free radical-scavenging and anti-oxidant properties of black berry against fluoride toxicity in rats. **2010**, *48*, 1999-2004.
2. Musa, A.; Shady, N.H.; Ahmed, S.R.; Alnusaire, T.S.; Sayed, A.M.; Alowaiesh, B.F.; Sabouni, I.; Al-Sanea, M.M.; Mostafa, E.M.; Youssif, K.A.; et al. Antiulcer Potential of Olea europea L. cv. Arbequina Leaf Extract Supported by Metabolic Profiling and Molecular Docking. **2021**, *10*, 644.
3. Shady, N.H.; Soltane, R.; Maher, S.A.; Saber, E.A.; Elrehany, M.A.; Mostafa, Y.A.; Sayed, A.M.; Abdelmohsen, U.R. Wound Healing and Antioxidant Capabilities of Zizyphus mauritiana Fruits: In-Vitro, In-Vivo, and Molecular Modeling Study. *Plants (Basel, Switzerland)* **2022**, *11*, doi:10.3390/plants11111392.
4. Sonboli, A.; Mojarrad, M.; Ebrahimi, S.N.; Enayat, S.J.I.J.o.P.R.I. Free radical scavenging activity and total phenolic content of methanolic extracts from male inflorescence of Salix aegyptiaca grown in Iran. **2010**, *9*, 293.
5. Alsenani, F.; Ashour, A.M.; Alzubaidi, M.A.; Azmy, A.F.; Hetta, M.H.; Abu-Baih, D.H.; Elrehany, M.A.; Zayed, A.; Sayed, A.M.; Abdelmohsen, U.R.J.M.d. Wound Healing

Metabolites from Peters’ Elephant-Nose Fish Oil: An In Vivo Investigation Supported by In Vitro and In Silico Studies. **2021**, *19*, 605.
